# Supplementary material for: Facile Fabrication of Oxygen-Releasing Tannylated Calcium Peroxide Nanoparticles
Source: Materials (Basel). 2020 Sep 1;13(17):3864. doi: 10.3390/ma13173864 (PMC7504056; doi:10.3390/ma13173864)
Supplement: Supplementary file 1 [file materials-13-03864-s001.pdf]

materials

Supplementary

Facile Fabrication of Oxygen-Releasing Tannylated Calcium Peroxide Nanoparticles

Ji Sun Park, Yeong Jun Song, Yong Geun Lim and Kyeongsoon Park *

Department of Systems Biotechnology, Chung-Ang University, Anseong, Gyeonggi 17546, Korea; park41917235@gmail.com (J.S.P.); rhksdn9502@naver.com (Y.J.S.); kgus0113@naver.com (Y.G.L.);

* Correspondence: kspark1223@cau.ac.kr; Tel.: +82-31-670-3357

Received: 16 July 2020; Accepted: 31 August 2020; Published: date

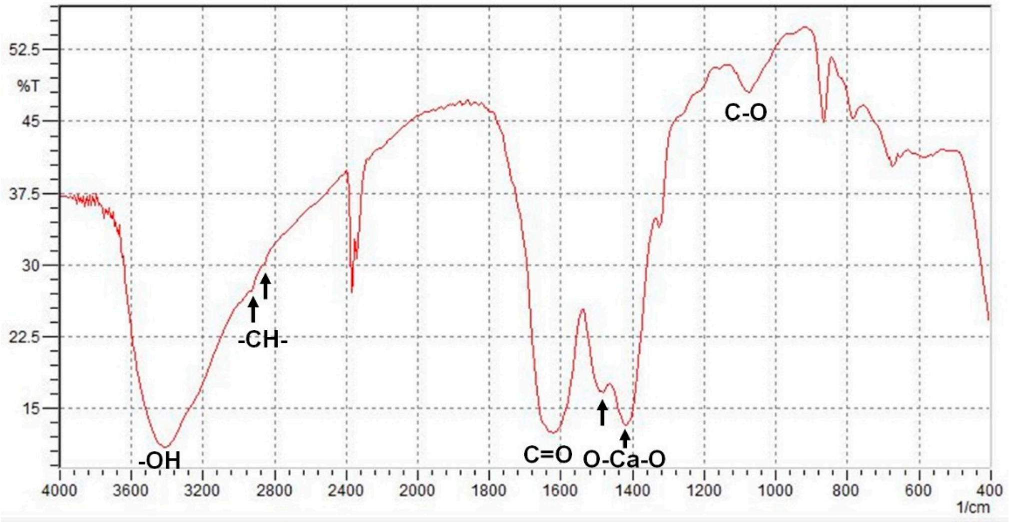

Figure S1. FT-IR spectrum of TA (10 mg)/CaO_2.

Table S1. Particle size analysis of the three TA/CaO_2 samples.

Sample	Mean Size (nm)	Z-Average Size (nm)*	PDI**
TA(10 mg)/CaO_2	263.4 ± 68.7	240.7	0.376
TA(25 mg)/CaO_2	228.7 ± 80.5	257.3	0.388
TA(50 mg)/CaO_2	221.9 ± 52.8	200.1	0.187

*Z-Average Size (nm): the intensity weighted harmonic mean size

**PDI: Polydispersity Index.

Table S2. Chemical composition of TA/CaO_2 from SEM-EDS analysis.

Sample	Elements			Total (%)
	C	O	Ca	
TA(10 mg)/CaO_2	7.86	43.71	48.43	100
TA(25 mg)/CaO_2	13.68	42.11	44.20	100
TA(50 mg)/CaO_2	21.65	42.28	36.07	100

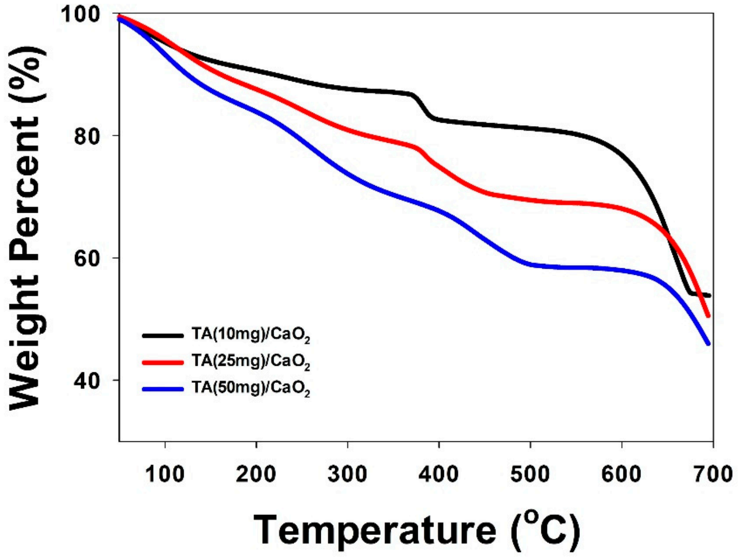

Figure S2. Thermal gravimetric analysis (TGA) curves of TA (10 mg)/CaO_2, (**b**) TA (25 mg)/CaO_2, and (**c**) TA (50 mg)/CaO_2.

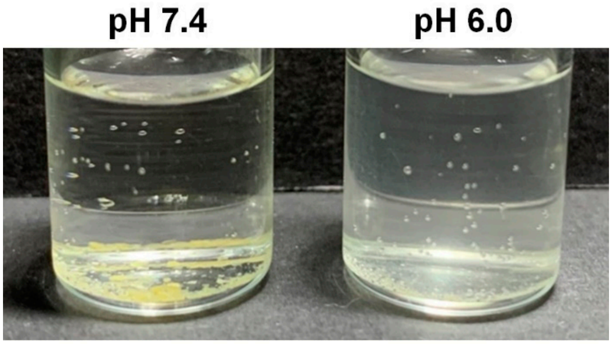

Figure S3. Dissolution test of TA/CaO_2 powder in PBS solutions of pH 7.4 and 6.0.

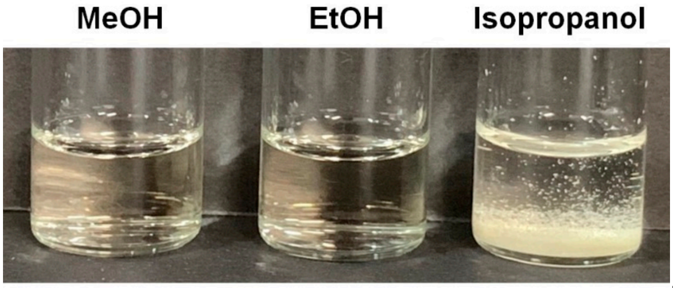

Figure S4. Solubility test of TA in three alcoholic solvents such as MeOH, EtOH, and isopropanol.

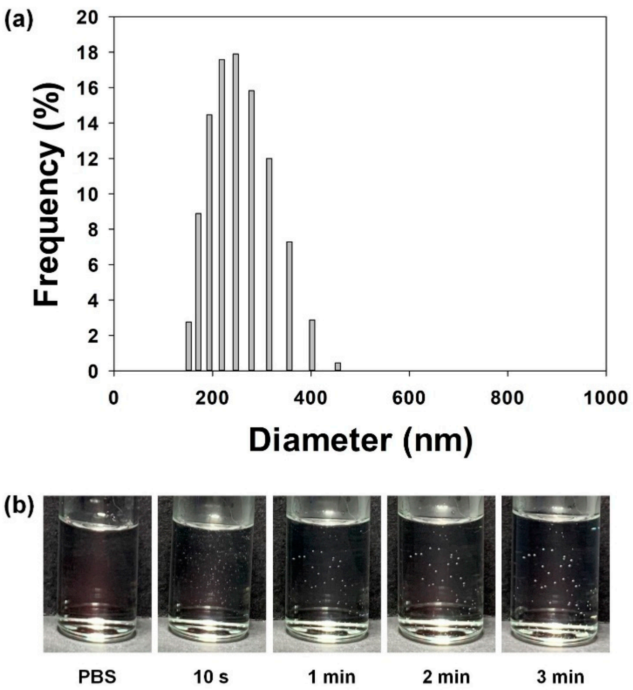

Figure S5. (**a**) Particle size distribution and (**b**) oxygen bubble generation of TA (10 mg)/CaO_2 nanoparticles synthesized in MeOH as a solvent.

© 2020 by the authors. Licensee MDPI, Basel, Switzerland. This article is an open access article distributed under the terms and conditions of the Creative Commons Attribution (CC BY) license (http://creativecommons.org/licenses/by/4.0/).
